# Non-contrast-enhanced magnetic resonance angiography of facial arteries for pre-operative evaluation of vascularized submental lymph node flaps

**DOI:** 10.1186/s12880-019-0368-7

**Published:** 2019-08-16

**Authors:** Ming-Chen Wu, Ming-Yi Hsu, Ren-Fu Shie, Ming-Huei Cheng, Fang-I Chu, Chien-Yuan Lin, Yui-Ping Fan, Sung-Yu Chu

**Affiliations:** 1 Department of Medical Imaging and Intervention, Chang Gung Memorial Hospital, Chang Gung University, Taoyuan, Taiwan; No. 5 Fuxing St., Guishan Dist, Taoyuan City, Taiwan; 2 Division of Reconstructive Microsurgery, Department of Plastic and Reconstructive Surgery, Chang Gung Memorial Hospital, Chang Gung University, Taoyuan, Taiwan; No. 5 Fuxing St., Guishan Dist, Taoyuan City, Taiwan; 3 Department of Statistics and Applied Probability, University of California, Santa Barbara, CA 93106-3110 USA; 4 Department of Radiation Oncology, University of California, 200 UCLA Medical Plaza, Suite B265, Los Angeles, CA 90095-6951 USA; 5 GE Healthcare, Taiwan; 6F, No.8, Min Sheng E. Rd., Sec. 3, Taipei, 10480 Taiwan

**Keywords:** 3D phase contrast-magnetic resonance angiography, Lymphedema, Vascularized submental lymph node flap

## Abstract

**Background:**

The aim of this study was to compare non-contrast-enhanced 3D phase contrast magnetic resonance angiography (3D PC-MRA) and conventional intravenous administration of contrast media, i.e., contrast-enhanced MRA (CE-MRA), to evaluate the courses of facial arteries for the preparation of vascularized submental lymph node flap (VSLN flap) transfer.

**Methods:**

The head and neck regions of 20 patients with limb lymphedema were imaged using a 3 T MRI scanner. To improve the evaluation of facial artery courses, MRA was fused with anatomical structures generated by high-resolution T1-weighted imaging. The diagnostic and image qualities of facial arteries for VSLN flap planning were independently rated by two radiologists. Interobserver agreement was evaluated using Cohen’s kappa. Differences between 3D PC-MRA and CE-MRA in terms of the diagnostic quality of facial arteries were evaluated using McNemar’s test.

**Results:**

Cohen’s kappa indicated fair to good interobserver agreement for the diagnostic and image qualities of the bilateral facial arteries. No significant difference in terms of the diagnostic quality of the left and right facial arteries between 3D PC-MRA and CE-MRA, respectively, was identified.

**Conclusions:**

Non-contrast 3D PC-MRA is a reliable method for the evaluation of facial artery courses prior to VSLN flap transfer and could serve as an alternative to CE-MRA for patients with renal insufficiency or severe adverse reactions to contrast media.

## Background

Lymphedema is a disease characterized by the abnormal collection of fluid and proteins in the interstitial space. The lymphedema could be due to congenital lymphatic dysplasia (primary lymphedema), anatomic obliteration (secondary lymphedema, such as after radical dissection, irradiation, repeated lymphangitis) or consequence of functional deficiency. Treatment of lymphedema includes conservative methods such as drug therapy, complex decongestive therapy, and more invasive methods, e.g., debulking surgery and microsurgery [1]. Recently, a vascularized submental lymph node flap (VSLN flap) with a facial vascular pedicle was developed for the treatment of limb lymphedema [2–7]. Contrast-enhanced (CE) magnetic resonance angiography (CE-MRA) and computed tomography angiography (CTA) are used to evaluate the main arteries of the maxillofacial region, particularly the facial arteries [8–12]. Non-CE-MRA also has been used to evaluate the carotid and intracranial main arteries [13, 14], but only one article to date has focused on the main arteries of the maxillofacial region. Sakai et al. proposed non-CE four-dimensional MRA with modified true fast imaging and steady-state free precession as well as a flow-sensitive alternating inversion recovery scheme for spin tagging of blood labeling sequences using a three Tesla (3 T) magnetic resonance imaging (MRI) system to evaluate 15 patients with head and neck tumors [15]. In this study, 81% of facial arteries were identified by two independent radiologists, but no other study has applied this method for the clinical evaluation of facial arteries.

In October 2010, a 3 T MRI system with a commercialized non-CE-MRA technique using three-dimensional phase contrast (PC) MRA (3D PC-MRA) was installed at our institute [16]. Unlike traditional PC-MRA, 3D PC-MRA is compatible with parallel imaging and uses a time-efficient elliptical k-space-filling technique to reduce scan time and influence patient motions, especially in children and the elderly. The aim of present study was to compare the performances of non-CE 3D PC-MRA and CE-MRA in terms of visualization of the facial arteries.

## Methods

### Patients

The study protocol was approved by the Chang Gung Medical Foundation Institutional Review Board (101–3481-B), and all participants provided an informed consent. Between January 2014 and April 2015, a total of 20 patients with limb lymphedema (1 male and 19 females; mean age: 54.9 years; age range: 2–70 years) as confirmed by lymphoscintigraphy preoperatively underwent MRI for VSLN flap transfer. None of the patients had a history of surgery for cancer of the head and neck region.

### MRI technique

All MRI examinations were performed using a 3 T clinical scanner (Discovery MR750; GE Healthcare, Milwaukee, WI, USA) equipped with an 8-channel brain coil for signal detection and body coil for radio frequency transmission. The MRI field-of-view (FOV) was 300 mm, and the tongue base area was placed at the center of FOV. The order of the scanning sequences were as follows: 3D fast spin-echo T1-weighted image (Volume-T1), 3D fast spin-echo T2-weighted image (Volume-T2), 3D PC-MRA, and CE-MRA. To better depict the courses of facial arteries, a facial anatomical reference was acquired by Volume-T1 in the sagittal view with a repetition time (TR) of 600 ms, echo time (TE) of 13 ms, FOV of 160 mm, and slab thickness of 12 mm. Similarly, to better identify the submandibular and submental lymph nodes (LNs), fat-saturated Volume-T2 was applied in the sagittal view with a TR of 2500 ms, TE of 76 ms, FOV of 160 mm, and slab thickness of 12 mm. Images were acquired by 3D PC-MRA and CE-MRA under matched slice coverage and resolution. The scanning parameters are summarized in Table 1. The acquisition time for CE-MRA was optimized, and trigger delay was set at 2 s following intravenous administration of 0.2 mmol/kg gadopentetate dimeglumine (Bayer Pharma AG, Wuppertal, Germany) to maximize the signal intensity of the branches of facial arteries in accordance with the findings of a MRA data trial conducted in our department. The stay duration of patients in the MRI room was approximately 25–30 min. Further, the acquisition time of 3D PC-MRA was 161 s, whereas that for CE-MRA was 119 s.

**Table 1:** Imaging parameters

	3D PC-MRA	CE-MRA
Field-of-view (mm)	300	300
Matrix size (mm)	352 × 224	352 × 224
Slice thickness (mm)	1.6	1.6
Repetition time (ms)	8.9	3.9
Echo time (ms)	3.9	1.4
Orientation of acquisition	Coronal	Coronal
Scan time (min:s)	02:41	01:59

*3D PC-MRA* 3D phase magnetic resonance angiography, *CE-MRA* Contrast-enhanced magnetic resonance angiography

### Data analysis

**Fig. 1:**
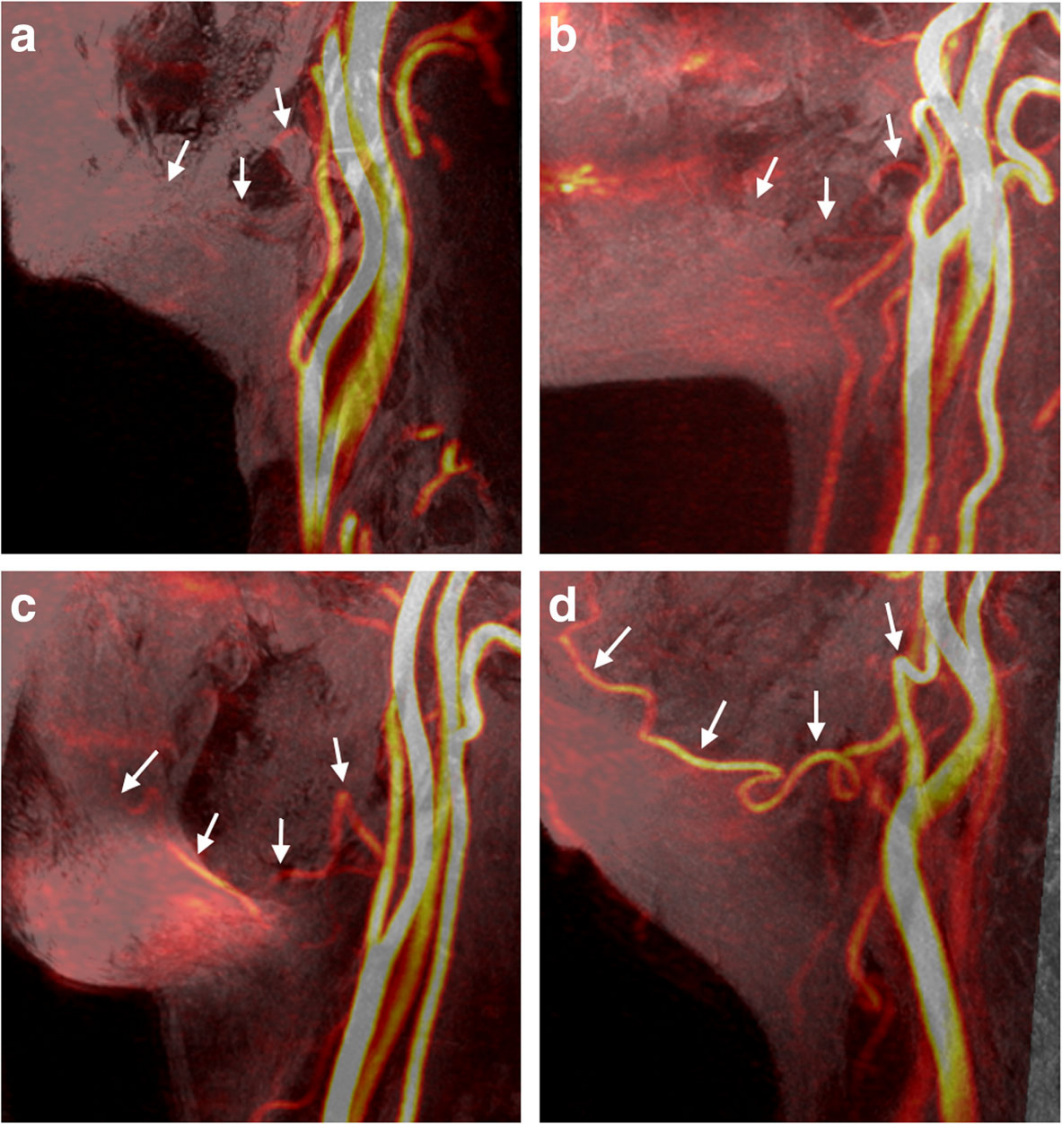
Examples of the image quality of 3D phase contrast magnetic resonance angiography (3D PC-MRA) to visualize the facial artery courses based on four-point score.0 = poor (**a**); 1 = questionable (**b**); 2 = adequate (**c**); and 3 = good (**d**). Green arrows indicate the facial artery course

Postprocessing of the MRI data was interactively performed using the integrated registration function of a standard workstation (AW VolumeShare 5; GE Healthcare, Waukesha, WI, USA). Slabs of 18 mm thickness were generated by maximum intensity projection (MIP) of Volume-T1 with 3D PC-MRA or CE-MRA. A total of 40 facial arteries of 20 anonymized patients were evaluated using 3D PC-MRA and CE-MRA. Further, MRA diagnosis and image quality for the visualization of facial arteries were assessed by two board-certified radiologists with 11- and 7-year experience in evaluating MRA images, respectively, who were blinded to patient information and imaging technique. The radiologists rated the diagnostic and image qualities of facial arteries using a four-point scale: 0 = poor quality of facial arteries with nondiagnostic quality (Fig. 1a); 1 = questionable or partial visualization of facial arteries with nondiagnostic quality (Fig. 1b); 2 = partial visualization of facial arteries with diagnostic quality (Fig. 1c); and 3 = good to excellent image with diagnostic quality (Fig. 1d).

### Statistical analysis

Statistical analysis were conducted to determine whether the diagnostic quality of facial arteries differed between 3D PC-MRA and CE-MRA. The obtained image quality score from the two radiologists was used as an indicator of image clarity obtained using 3D PC-MRA and CE-MRA. Diagnostic quality was further denoted as a categorical variable wherein an image quality score of 2 or 3 was considered diagnostic and a score of 0 or 1 was considered nondiagnostic. Interobserver agreement between the two radiologists was evaluated using Cohen’s kappa statistic [17]; Fleiss’s arbitrary guidelines characterize kappas > 0.75 as excellent, 0.40–0.75 as fair to good, and < 0.40 as poor [18]. For the facial arteries of the right and left sides, respectively, difference in terms of diagnostic quality between 3D PC-MRA and CE-MRA was assessed using McNemar’s test. All analysis were conducted using R software [19]. A *p*-value of < 0.05 was considered statistically significant.

## Results

**Fig. 2:**
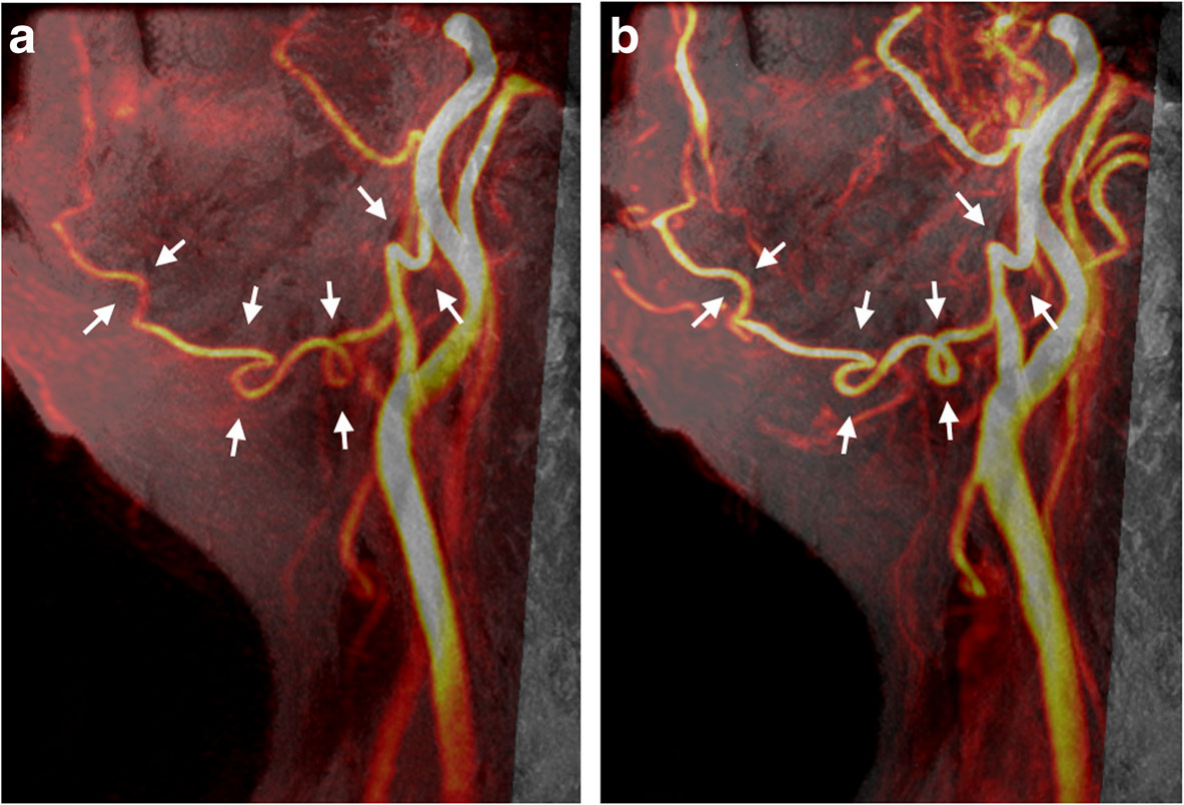
Left lateral view by maximum intensity projection (MIP) from Volume-T1 fused with (**a**) 3D PC-MRA and (**b**) contrast-enhanced MRA (CE-MRA) of a 70-year-old female with lymphedema

**Fig. 3:**
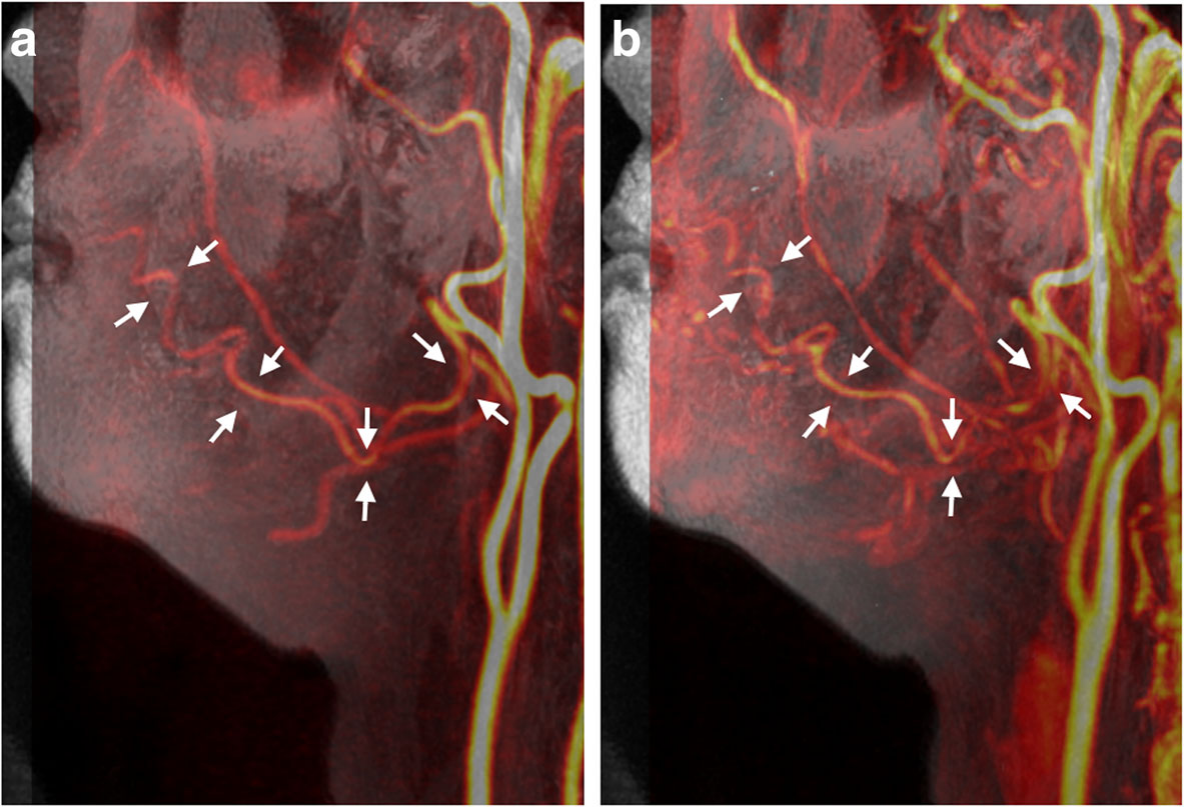
Left lateral view by MIP from Volume-T1 fused with (**a**) 3D PC-MRA and (**b**) CE-MRA of a 51-year-old female with lymphedema. Visualization of the facial artery may be difficult with CE-MRA due to the contamination of contrast enhancement of neighboring soft tissues and veins

**Fig. 4:**
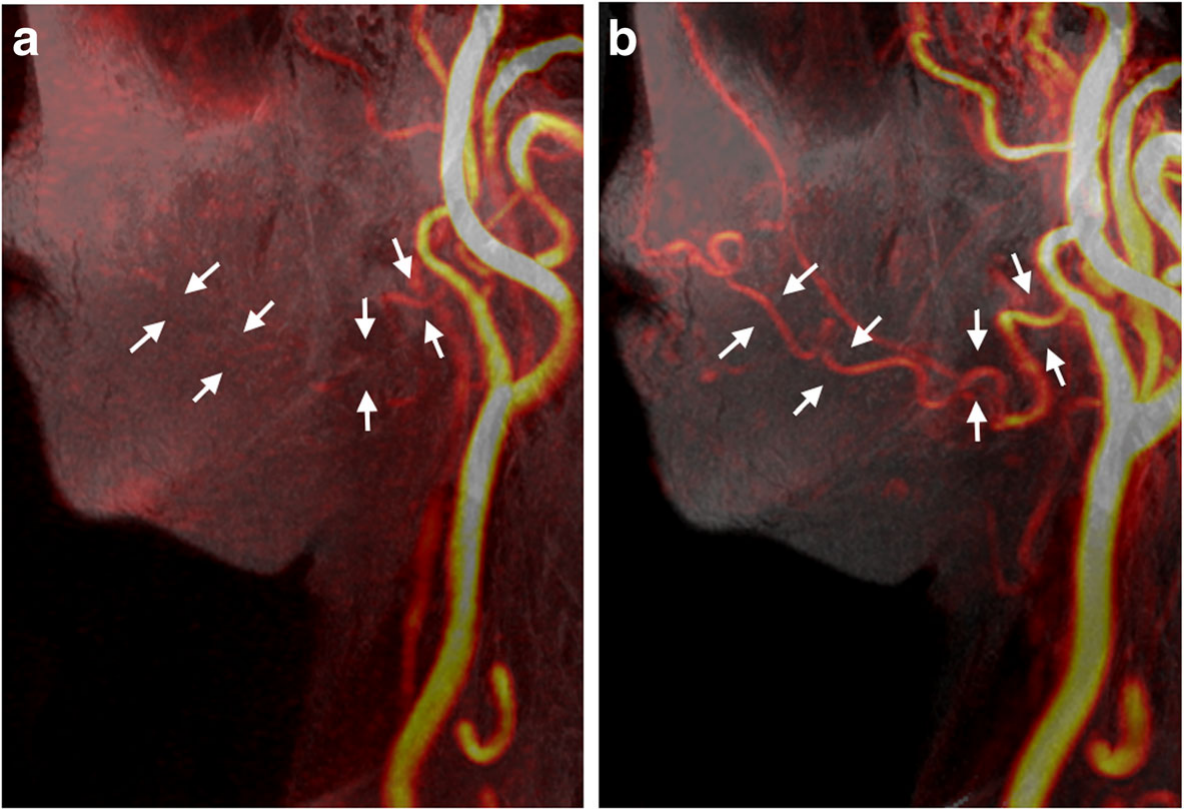
Left lateral view by MIP from Volume-T1 fused with (**a**) 3D PC-MRA and (**b**) CE-MRA of a 48-year-old female with lymphedema. The signal of the facial artery may be lost with 3D PC-MRA due to flow change

All 40 facial arteries from 20 patients were successfully imaged using 3D PC-MRA and CE-MRA. Of these, 15 arteries (37.5%) imaged using 3D PC-MRA (Fig. 2a) and 21 (52.5%) using CE-MRA (Fig. 2b) were exactly delineated by both radiologists. Because the enhancement of facial arteries is associated with contrast media (CM)-induced T1-shortening of the blood, it may be sometimes difficult to differentiate the small arteries from neighboring tissues due to CM contamination with CE-MRA compared with 3D PC-MRA (Fig. 3). For 1 of the 20 patients, the signals of facial arteries were lost with 3D PC-MRA but not with CE-MRA (Fig. 4).

The radiologists rated vascular morphology by MIP of Volume-T1 fused with 3D PC-MRA or CE-MRA using a four-point scale. The results are summarized in Table 2. Regarding the image quality of the right and left facial arteries on the images acquired with 3D PC-MRA, the estimated Cohen’s kappa were 0.67 and 0.55, respectively, indicating fair to good agreement. As shown by the summary of McNemar’s test results in Table 3, no significant difference in terms of diagnostic quality between the images acquired with 3D PC-MRA and those acquired with CE-MRA was identified. The image quality scores are summarized in Table 4.

**Table 2:** Image and diagnostic quality scores by two ratersImage quality score, 0–3

Patient	Image Quality								Diagnostic Quality							
	Right				Left				Right				Left			
	3D PC-MRA Rater 1	3D PC-MRA Rater 2	CE- MRA Rater 1	CE-MRA Rater 2	3D PC-MRA Rater 1	3D PC-MRA Rater 2	CE-MRA Rater 1	CE-MRA Rater 2	3D PC-MRA Rater 1	3D PC-MRA Rater 2	CE-MRA Rater 1	CE-MRA Rater 2	3D PC-MRA Rater 1	3D PC-MRA Rater 2	CE-MRA Rater 1	CE-MRA Rater 2
1	2	2	3	2	1	1	3	3	D	D	D	D	N	N	D	D
2	3	3	3	3	3	3	3	3	D	D	D	D	D	D	D	D
3	2	3	3	3	2	2	2	2	D	D	D	D	D	D	D	D
4	0	0	3	2	1	2	3	2	N	N	D	D	N	D	D	D
5	2	2	3	3	2	2	3	2	D	D	D	D	D	D	D	D
6	3	3	3	3	3	3	3	3	D	D	D	D	D	D	D	D
7	3	3	3	3	2	2	3	3	D	D	D	D	D	D	D	D
8	2	1	2	2	3	2	3	3	D	N	D	D	D	D	D	D
9	2	2	1	2	2	2	2	2	D	D	N	D	D	D	D	D
10	3	3	3	3	3	3	3	3	D	D	D	D	D	D	D	D
11	3	3	0	0	3	2	0	0	D	D	N	N	D	D	N	N
12	3	3	3	3	2	2	2	2	D	D	D	D	D	D	D	D
13	2	1	2	2	2	2	3	3	D	N	D	D	D	D	D	D
14	3	3	3	3	1	2	3	2	D	D	D	D	N	D	D	D
15	3	3	2	2	0	0	0	0	D	D	D	D	N	N	N	N
16	2	2	3	3	1	1	3	2	D	D	D	D	N	N	D	D
17	2	1	3	3	2	1	3	2	D	N	D	D	D	N	D	D
18	3	3	3	3	3	2	3	2	D	D	D	D	D	D	D	D
19	3	3	3	3	3	3	3	3	D	D	D	D	D	D	D	D
20	2	2	3	3	3	3	3	3	D	D	D	D	D	D	D	D
Cohen’s kappa	0.67		0.68		0.56		0.49		0.35		0.64		0.571		1	

Diagnostic score, N: nondiagnostic; D: diagnostic

*3D PC-MRA* 3D phase magnetic resonance angiography, *CE-MRA* Contrast-enhanced magnetic resonance angiography

**Table 3:** Obtained *p*-values using McNemar’s test to access differences between 3D phase magnetic resonance angiography and contrast-enhanced magnetic resonance angiography

	Rater 1	Rater 2
Right side	1	0.37
Left side	0.37	0.62

**Table 4:** Summary of image quality scores of the bilateral facial arteries by the two raters

SCORE	Right facial artery				Left facial artery			
	3D PC-MRA Rater 1	3D PC-MRA Rater 2	CE-MRA Rater 1	CE-MRA Rater 2	3D PC-MRA Rater 1	3D PC-MRA Rater 2	CE-MRA Rater 1	CE-MRA Rater 2
0	1	1	1	1	1	1	2	2
1	0	3	1	0	4	3	0	0
2	9	5	3	6	7	11	3	9
3	10 (50%)	11 (55%)	15 (75%)	13 (65%)	8 (40%)	5 (25%)	15 (75%)	9 (45%)

3D PC-MRA: 3D phase magnetic resonance angiography, CE-MRA: contrast-enhanced magnetic resonance angiography

## Discussion

To date, a limited number of articles have proposed the use of sonography, computed tomography, and MRI to evaluate the submandibular and submental LNs within VSLN flap [2, 20–22], but no study has explored the relationship between the submandibular glands and facial arteries during VSLN flap transfer. Pre-operative evaluation of LNs within VSLN flap and the courses of facial arteries prior to VSLN flap transfer is important, especially to determine the relationship while planning the ligation of branches to the submandibular glands and to reduce the surgical duration required to prepare facial arteries [23, 24]. The use of CM-enhanced CTA or MRA could facilitate the elucidation of the relationship between the submandibular glands and facial arteries prior to VSLN flap transfer. Although the cost and examination time of CTA are lower than those of MRA, the latter is more favorable owing to the lack of radiation exposure [25] and no risk of CM-induced nephropathy [26–28].

With a proper setting of acquisition timing following the administration of intravenous CM injection, CE-MRA is considered a promising method for the visualization of facial arteries [2, 8–11]. However, two factors should be considered when applying CE-MRA: complication of the delineation of facial arteries due to CM contamination of neighboring tissues (Fig. 3b) and potential risk of nephrogenic systemic fibrosis following the administration of gadolinium-based CM in patients with renal insufficiency [27, 29, 30]. Non-CE-MRA is a viable alternative method for the visualization of the main arteries in the neck region. 3D PC-MRA based on blood flow velocity has minimal background tissue intensity and demonstrates comparable results to CE-MRA in terms of the visualization of facial arteries (Figs. 2 and 3). In this study, there was no significant difference between CE-MRA and 3D PC-MRA in terms of the diagnostic abilities of facial arteries. The signal loss of facial arteries with 3D PC-MRA in one patient (Fig. 4) may be attributable to flow change, suggesting that an improper velocity encoding (VENC) value was used. An automatic method for rapid determination of a suitable range of the VENC values of specific vessels would be helpful when applying 3D PC-MRA to avoid blood flow perturbations.

Regarding image quality, on an average, 65 and 42.5% of the images for bilateral facial arteries with CE-MRA and 3D PC-MRA, respectively, had a score of at least 3, suggesting that image quality may be better with CE-MRA. However, further investigations with larger cohorts are necessary to identify significant differences in terms of image quality scores.

Despite the potential differences in terms of image quality between CE-MRA and 3D PC-MRA, the difference in terms of diagnostic quality between both methods was consistently insignificant for the bilateral facial arteries. Three dimensional PC-MRA, a noninvasive method, has a lower cost and no risk of adverse reactions from CM; thus, radiologists should first consider 3D PC-MRA for pre-operative planning of VSLN flap transfer. With no intravenous administration of CM, 3D PC-MRA can be immediately repeated if image quality is unsatisfactory and can relieve anxiety and stress among children during the placement of an intravenous catheter. CE-MRA should be considered a bailout method when the results of 3D PC-MRA are consistently unsatisfactory. On the other hand, 3D PC-MRA could serve as an alternative method for CE-MRA failure due to motion artifacts or missed optimal acquisition timing.

There were two limitations to this study that should be addressed. First, the branches and perforators of facial arteries were not evaluated. CTA can be used for the pre-operative evaluation of perforators during flap transfer [31, 32], whereas CE-MRA with a small FOV is better for imaging facial arteries and branches and perforators. However, the signal-to-noise ratio of image quality due to a small FOV remains problematic, and scan time increases due to repeated studies of the right and left facial arteries. To the best of our knowledge, no study has yet examined the efficiency of non-CE-MRA in the evaluation of small vessels, such as perforators, or the branches of the main arteries of the maxillofacial region. Thus, the next stage is to image the branches and perforators with 3D PC-MRA. Second, the sample size of this study was relatively small (i.e., 20 patients); therefore, further evaluations using larger population size is necessary to verify these results.

## Conclusions

3D PC-MRA is a reliable method for the evaluation of facial artery course during the planning of VSLN flap transfer. 3D PC-MRA provides similar diagnostic accuracy and acceptable image quality compared to CE-MRA, without the risk of adverse drug reactions due to CM or nephrogenic systemic fibrosis. Consequently, 3D PC-MRA is expected to be widely applied to improve the diagnostic value of the visualization of small arterial branches.

## Data Availability

The datasets used and/or analyzed during the current study are available from the corresponding author on reasonable request.

## Abbreviations

3D PC-MRA: Three-dimensional phase contrast magnetic resonance angiography
3T: Three Tesla
CE: Contrast-enhanced
CE-MRA: Contrast-enhanced magnetic resonance angiography
CM: Contrast media
CTA: Contrast media-enhanced computed tomography angiography
FOV: Field-of-view
LNs: Lymph nodes
MIP: Maximum intensity projection
MRI: Magnetic resonance imaging
TE: Echo time
TR: Repetition time
VENC: Velocity encoding
Volume-T1: Three-dimensional fast spin-echo T1-weighted image
Volume-T2: Three-dimensional fast spin-echo T2-weighted image
VSLN flap: Vascularized submental lymph node flap
